# Analysis of the genetic and phylogenetic context of *Escherichia coli* O77g:H18 associated with clustered cases of HUS in France in 2025

**DOI:** 10.1128/aem.02449-25

**Published:** 2026-04-24

**Authors:** Fabien Vorimore, Mai-Lan Tran, Aurélie Cointe, Justine de Larminat, Philippe Bidet, Stéphane Bonacorsi, Carolina Silva Nodari, François-Xavier Weill, Patrick Fach, Sabine Delannoy

**Affiliations:** 1 Laboratory for Food Safety, Unit of “Pathogenic E. coli” (COLiPATH) and Genomics platform “IdentyPath” (IDPA), Anses (The French Agency for Food, Environmental and Occupational Health and Safety) 55036, Maisons-Alfort, France; 2 Service de Microbiologie, Centre National de Référence Escherichia coli, AP-HP, Hôpital Robert-Debré (DMU BioGeM), F-75019, Université Paris Cité, IAME, UMR 1137, INSERM 27102, Paris, France; 3 Institut Pasteur, Université Paris Cité, Unité des Bactéries pathogènes entériques, Centre National de Référence des Escherichia coli, Shigella et Salmonella, Paris, France; Colorado School of Mines, Golden, Colorado, USA

**Keywords:** Shiga toxin-producing *E. coli *STEC, enterohemorrhagic *E. coli *EHEC, O77g:H18, stx2d, putative enterotoxin, IncFIA/FIB plasmid

## Abstract

In January 2025, Santé publique France detected several cases of hemolytic uremic syndrome (HUS) in adults caused by a rare serotype of Shiga toxin-producing *Escherichia coli*, STEC O77g:H18. Epidemiological and traceability investigations linked the consumption of raw-milk cheese to these cases. The outbreak strain belonged to sequence type 69 (ST69) and harbored a Shiga toxin (stx) subtype *stx2d* located on a unique prophage. Putative virulence genes *cdt-VABC*, *hlyE*, *fimH*, and *lpfA* were detected; however, STEC virulence genes (*eae* and *aggR*) were absent. Phylogenetic analysis of *E. coli* O77g strains from public databases (Enterobase and NCBI) placed the outbreak strain within a cluster of clinical *E. coli* O77g:H18, ST69, *stx2d* strains isolated between 2012 and 2025, including one linked to a HUS case in France and three in Australia. This strain, distinct from O77g:H18 strains previously isolated from dairy products, is therefore not a new lineage. The closest cluster contains eight *stx*-negative strains isolated from poultry between 1990 and 2024 in Europe and North America. Differences in virulence gene composition between the “outbreak cluster” and the “poultry cluster” suggest a common ancestor that has diverged over time. The “outbreak cluster” is characterized by strains carrying an IncFIA/FIB plasmid encoding a putative enterotoxin gene (*elt*) absent in other *E. coli* strains in GenBank. We developed qPCR assays specific for the putative enterotoxin and O77g O-group. Combined with a PCR for *stx2*, these assays allow rapid screening of suspect unpasteurized cheese for the outbreak strain.

**IMPORTANCE:**

The emergence of hybrid pathotypes of Shiga toxin-producing *Escherichia coli* (STEC) has been of importance in recent years in public health. This outbreak, related to a rare serotype of STEC and involving an enterotoxigenic *E. coli* (ETEC)/STEC hybrid, highlights the necessity of phylogenetic analyses to investigate the origin of contamination with such strains. Our study suggests a common ancestor between the outbreak strain and strains isolated from poultry. We also provided molecular tools to target specifically this outbreak strain and help in future epidemiological investigations.

## INTRODUCTION

Shiga toxin-producing *Escherichia coli* (STEC) are commonly found in the intestine of wild and domestic ruminants. Excretion of STEC with animal feces results in a broad contamination of food and the environment. Search for natural STEC reservoirs and for *E. coli* serotypes associated with Stx production intensified with the development of molecular detection and characterization methods. Today, it is known that serologically very diverse STEC types are present not only in ruminants but also in many species of mammals, as well as birds, insects, fish, and mollusks (1). Insects and birds play an important role in disseminating STEC over long distances, thus allowing its spread across large geographical areas (1). A recent study on the shedding dynamics of foodborne pathogens by wild birds on farmlands in California found identical STEC strains (O157, O26, and O103) shared episodically among birds and between wild geese and free-range cattle, suggesting a common source of contamination in pre-harvest environments and potential transmission between species (2).

Various serotypes of STEC were found to persist in the environment. Once in the environment, STEC may survive in soil and water for weeks and months, depending on biotic factors and the physicochemical conditions in general (3). The occurrence of free *stx* phage particles in the environment also contributes to the dissemination of STEC (4). Transfer of *stx* genes to *E. coli* was found to occur by free *stx* phages in food and water, giving numerous possibilities for the generation and spread of STEC strains (5). Thus, evidence for horizontal transfer of STEC strains and recontamination of animals has been clearly documented in the literature (6). Direct and indirect transmission of STEC between animals and from animals to humans may occur (1, 7). Contamination of food of animal origin, like dairy products, occurs frequently at the critical stages of food production, such as milking. Accordingly, numerous types of STEC are found in raw milk products (8–10).

In humans, STEC could be associated with severe infections like bloody diarrhea and hemolytic uremic syndrome (HUS). More than 400 STEC serotypes have been isolated from sporadic and epidemic cases, but only a few, known as the top 7 (O157:H7, O26:H11, O111:H8, O103:H2, O145:H28, O121:H19, and O45:H2), are responsible for the majority of the infection cases (11). These strains harbor the *stx* gene(s) and the *eae* gene encoding intimin, an adhesion factor to the intestinal mucosa that was first identified in enteropathogenic *E. coli*. In addition to these typical *eae*-positive STECs associated with most of the severe infections related to raw milk and meat products, some atypical STEC strains isolated in humans encode *stx* genes but do not carry the *eae* gene. Clinically significant atypical STECs belong mainly to O-groups O91, O171, O174, O148, O146, O128, O113, and O104 (12). However, as they are usually not associated with large foodborne outbreaks, they are not currently monitored in food from bovine origin. For routine testing of foods, the reference method (13) prioritizes a hierarchical surveillance system focusing on high-risk STEC rather than all STEC. In a previous study, we showed that STEC prevalence in raw milk and unpasteurized cheese was 21.5% and 30.5%, respectively, and that 34 STEC strains isolated from these dairy samples evidenced a large diversity of serotypes and virulence factors complicating consumer risk assessments (8). More recently, in a study on beef samples (14), we showed that *eae*-negative STEC appears extremely predominant in beef samples, whereas it remains very rarely associated with STEC infections in France (12). These studies show that there is no correlation between clinical epidemiological data and those from the beef and dairy industries. Such discordance confirms in fact that the virulence of STEC remains difficult to define and predict with high confidence, being certainly multifactorial, combining virulence traits of STEC strains and host factors. Indeed, vulnerable populations like infants, the elderly, and immunocompromised people seem to be particularly affected.

Serotyping remains an essential and widely used typing method in the study and epidemiology of *E. coli* strains. In *E. coli*, most genes required for O-antigen biosynthesis are grouped in a single cluster, the O-antigen cluster (O-AGC), the composition of which is specific for each serogroup. In particular, the sequence of the genes involved in O-unit translocation and chain synthesis (*wzx*/*wzy* or *wzm*/*wzt*) is somewhat unique for each serogroup and can be used as targets for the identification of O serogroups via molecular approaches in genoserotyping (15, 16). Several serogroups traditionally distinguished by sero-agglutination have the same O-AGC and cannot be differentiated by genoserotyping, including O17, O44, O73, O77, and O106, collectively referred to as O77g (15, 16). Indeed, these serogroups share a common sugar backbone encoded by the same O-AGC, and the distinction arises from post-polymerization modifications of the O-antigens mediated by glucosyltransferases encoded outside of the O-AGC. In contrast to the O77 antigen, which lacks substitutions, the other O-antigens in this group are distinguished by the presence of one or two glucose side branches attached at different positions along the backbone (17).

In 2023, Anses proposed a categorization of STEC strains based on French clinical epidemiological and microbiological data and international literature. Among four defined groups, group II strains (*stx2a* and/or *stx2d*-positive, *eae*-negative, and *aggR-*negative) accounted for only 5% of the notified HUS and 6% of the notified bloody diarrhea from 2017 to 2021 in France (12). During this period, among the rare clinical isolates collected in group II, a few clinical isolates of STEC O77g:H18 associated with HUS were reported in France (National reference center for *E. coli*, personal communication).

However, since January 2025, the French health authorities investigated several cases of HUS that occurred in adults only and related to STEC O77g:H18 (18). Cases were confirmed by the National Reference Center for *Escherichia coli* (Institut Pasteur and Robert Debré Hospital, Paris). The outbreak strain, identified as sequence type 69 (ST69), HC5_326896 (EnteroBase HierCC-cgMLST scheme), *stx*2d positive and *eae* negative, was linked to raw milk soft cheese consumption. In France, it could be noticed that serotype O77g:H18 has rarely been detected in milk products (8), while being on the contrary reported in many occasions in bird and poultry (19, 20). In addition to the French clinical isolates, the National Health Service in Scotland reported at the same period one clinical strain (003-SME250038-I) having the same serotype and virulence profile that was isolated from a patient who consumed French cheese ordered online (Holmes personal communication). This study aims to investigate the genetic and phylogenetic context of *E. coli* O77g:H18 responsible for clustered cases of HUS in France in 2025. The second objective was to develop a qPCR test targeting a putative enterotoxin gene (*elt*) unique to the French outbreak strain STEC O77g:H18-ST69, *stx2d* to implement a rapid screening of unpasteurized cheese.

## MATERIALS AND METHODS

### Data set of bacterial strains used in this study

In this study, we sequenced 15 *E. coli* strains from our collection belonging to serogroups O17, O44, O73, O77, O106, and collectively referred to as O77g. The same O-antigen cluster is found in serogroups O17, O44, O106, O73, and O77 (15, 16). We named here by O77g strains belonging to the genetic group of strains from that O-group cluster. They included 12 strains isolated in Germany (8 strains isolated from humans [21] and 4 strains isolated from poultry [22]), 2 *E. coli* O77g strains isolated in France (1 strain isolated from the environment and 1 isolated from a dairy product [8]), as well as the *E. coli* O17:H18 reference strain K12a (23, 24). Assemblies have been deposited in the European Nucleotide Archive database under the bioproject PRJEB89238. We also collected *E. coli* O77g WGS data available in the National Center for Biotechnology Information (NCBI, https://www.ncbi.nlm.nih.gov/) and EnteroBase (http://enterobase.warwick.ac.uk/species/ecoli) databases. We downloaded a subset of *E. coli* O77g assembled genome sequences from Enterobase (*n* = 204, access date 25 February 2025, https://enterobase.warwick.ac.uk/species/ecoli/search_strains?query=workspace:145591) and NCBI (*n* = 6). We selected preferentially genomes with the H18 antigen and with the most complete metadata, while mixing different sources and collection years. In addition, we included four assembled genome sequences from STEC O77g:H18 strains (202500190, 202500196, 202500057, and 202500194) isolated in France in 2025 from confirmed cases of the *E. coli* O77g outbreak, which are also available in Enterobase (18). Thus, a total of 229 *E. coli* O77g strains were analyzed (listed in File S1). This data set aimed at providing an overview of the genetic context of the STEC O77g:H18 strains.

### Genome sequencing and assembly

Genomic DNA was extracted and purified from BHI cultures (1 mL) using the DNeasy blood and tissue kit (Qiagen) following the manufacturer’s instructions. Strains ECA64 and LP26-E1-261 were sequenced with a MiSeq (Illumina). Libraries were prepared from 1 ng of gDNA using the Nextera XT DNA Library Preparation Kit (Illumina Inc.) following the manufacturer’s instructions. Libraries were sequenced using the MiSeq Reagent Kit v2 (2 × 150 bp; Illumina Inc.) on a MiSeq System. The other 13 strains were sequenced using a MinION MK1C with R10.4.1 flow cells (Oxford Nanopore Technologies). The MinION libraries were prepared with 200 ng DNA using the rapid barcoding kit (SQK-RBK114.24, Oxford Nanopore Technologies) according to the manufacturer’s instructions. The MinION read data were generated in pod5 format using MinKNOW v24.11.8. The reads were basecalled and demultiplexed using dorado v0.8.1. *De novo* assembly of strain K12a was performed using trycycler v0.5.5. *De novo* assembly of the other strains was performed using flye v2.9.4 with the --nano-hq parameter.

### Phylogenetic analyses

Core genome multilocus sequence typing (cgMLST) analysis was performed as described in reference 25 using chewBBACA v3.3.10 and the Innuendo *E. coli* cgMLST scheme based on 2360 loci (26, 27). A custom Python script (File S2) was used to remove samples with <90% of alleles identified and remove loci identified in <90% of samples. The ExtractCgMLST module of chewBBACA was used to replace all non-numerical values in the resulting table with 0. A neighbor-joining tree was reconstructed from the cgMLST profiles using grapetree v2.1 (https://github.com/achtman-lab/GrapeTree [28]) and displayed using microreact (https://microreact.org/).

### Strain characterization

We confirmed the strains’ serotypes using Abricate (https://github.com/tseemann/abricate) with the Ecoh database (29). We determined their content in plasmids, virulence genes (VGs), and antimicrobial resistance (AMR) genes using Abricate with the PlasmidFinder (30), VirulenceFinder (31), and ResFinder databases (32). Additionally, we determined their *stx* subtype using Abricate and the stx database (https://bitbucket.org/genomicepidemiology/virulencefinder_db/src/master/stx.fsa). Their sequence types (STs) were confirmed using MLST v2.0 (with the *E. coli* #1 MLST scheme and database version 2023-06-19) on the CGE website (https://www.genomicepidemiology.org/ [33, 34]). The phylogroup was determined using EzClermont v0.7.0 (https://github.com/nickp60/EzClermont).

The VGs investigated included genes encoding toxins/hemolysins (*stx*, *cdt-VABC*, *estb-STb2*, *ehxA*, *hlyA*, *hlyE*, and *hlyF*), adhesins/fimbriae (*fimH*, *eae*, *iha*, *lpfA*, *efa1*, *paa*, and *papA*), type III secretion system effectors (*espY1*, *espY2*, *espY3*, *espY4*, *espA*, *espA*, *espB*, *espC*, *espD*, *espF*, *espG*, *espH*, *espI*, *espJ*, *espK*, *espL1*, *espL2*, *espM1*, *espM2*, *espN*, *espR1*, *espV*, *espW*, *espX1*, *espX4*, *espX5*, *espP*, *espZ*, *map*, *nleA*, *nleB1*, *nleB2*, *nleC*, *nleD*, *nleE*, *nleF*, *nleG7*, *nleH1*, *nleH2*, *nleL*, *tccp*, *tir*, and *ospG*), type II secretion system (*gspCDEFGHIJKLM*; referred to as the *gsp* cluster), aerobactin (*iutA* and *iucABCD*), yersiniabactin (*ybtAEPQSTUX* [referred to as the *ybt* cluster], *irp1*, *irp2*, and *fyuA*), salmochelin (*iroN*) temperature-sensitive hemagglutinin (*tsh*), cyclomodulin (*cif*), virulence features of avian pathogenic *E. coli* (APEC; *iss* and *sitA*), and *aggR* (transcriptional regulator of Enteroaggregative *E. coli*).

### Stx phage analysis

Prophage regions in strain M11957 were determined with Phastest v1.0.1 (35) in Proksee (https://proksee.ca). The gene adjacent to the integrase was designated as the phage insertion site (36). The *stx*-phage region was extracted from the closed genome sequences of strains M00057, M11957, and M7424 (accession SAMN16077358, SAMN16077359, and SAMN16435111), compared with various *stx*-phages (37) (File S3) using a *k*-mer-based approach (*k* = 15), and a UPGMA tree was constructed. The structures of the Stx prophages were compared using Mauve (38) and Proksee.

### Real-time PCR assays

One sample collected from a batch of unpasteurized cheese suspected to be associated with the STEC O77g:H18 French outbreak was enriched overnight at 37°C in buffered peptone water, and DNA was extracted using DNeasy Blood and Tissue kit (Qiagen) following the manufacturer’s protocol. Additionally, 45 DNA extracts from cow raw milk cheese samples obtained in a previous study (39) were included in this study to evaluate the genetic marker occurrence in unrelated samples.

Nucleotide sequence data from the previously published sequence of the lipopolysaccharide O77g antigen gene cluster (16) were used for designing primers and a FAM-labeled TaqMan qPCR probe for specific detection of *E. coli* O77g-antigen. In a similar way, we developed two qPCR assays for the specific detection of both sub-units of the putative enterotoxin (*elt*) found on the IncFIA/FIB plasmid of the French outbreak clone (File S4). Real-time PCR assays were performed with a CFX96 real-time PCR detection system (Bio-Rad). Reactions were performed in a final volume of 50 µL, containing 1× PerfeCTa qPCR ToughMix (Quanta Bio), 300 nM of each forward primer, reverse primer, and TaqMan probe, and 5 µL of DNA extract. PCR cycling conditions comprised of 95°C for 10 min, followed by 40 cycles of 95°C for 15 s and 60°C for 60 s, and a final step at 40°C for 30 s.

## RESULTS

### Phylogenetic analyses of *E. coli* O77g:H18, ST69

**Fig 1:**
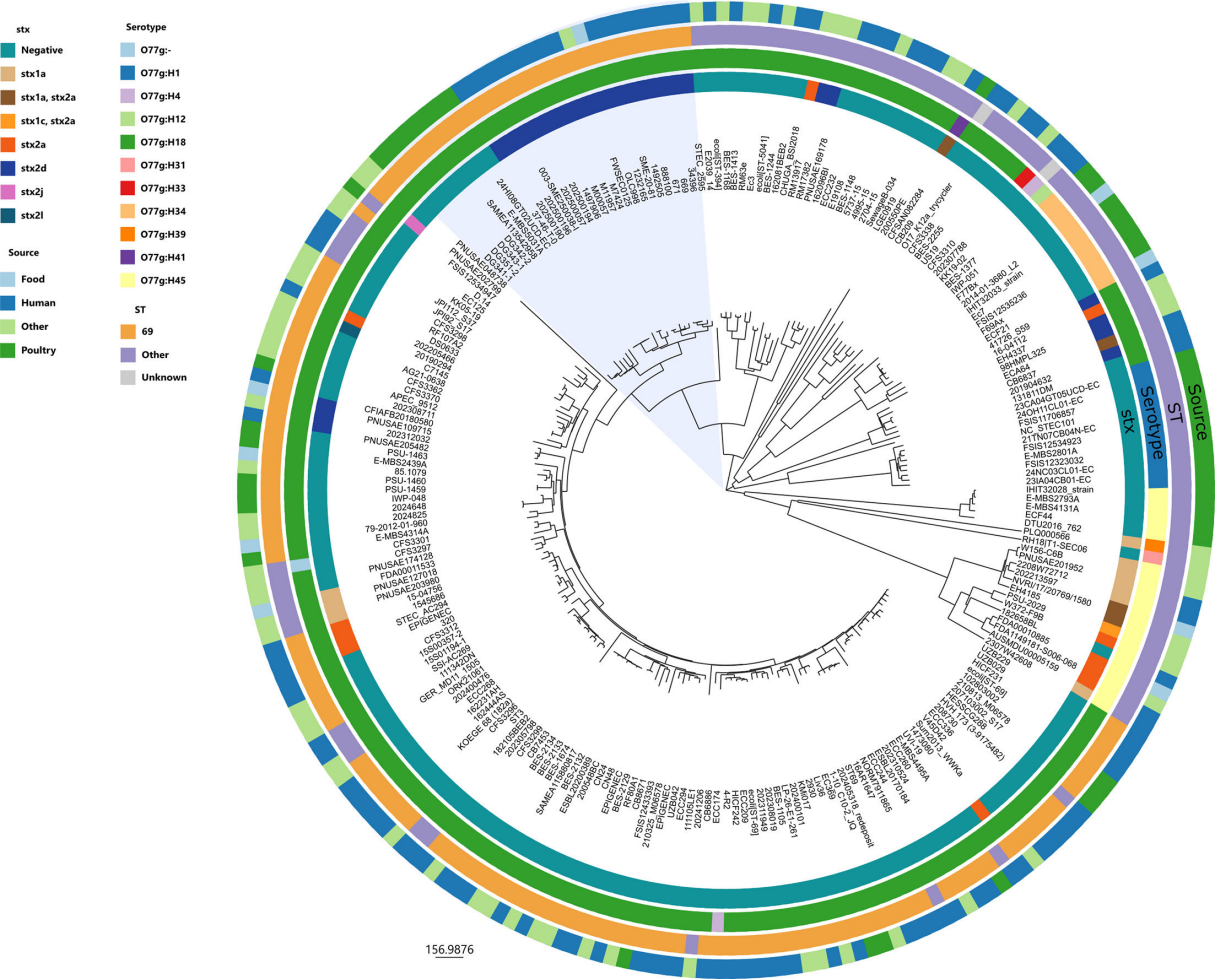
Neighbor-joining tree based on the core genome multilocus sequence typing (cgMLST) of *E. coli* isolates. The outer ring represents the source of the sample, the second ring represents the sequence type (ST), the third ring represents the serotype, and the inner ring represents the presence of a Shiga toxin (*stx*). A slice of the tree was colored in blue and represents the “outbreak cluster,” including the neighbor poultry clade.

A total of 229 *E. coli* O77g strains were analyzed in this study. The phylogenetic tree was reconstructed based on the cgMLST analysis (Fig. 1) and shows distinct clades relative to the ST, serotypes, presence of a Shiga toxin (stx) gene, and the sources of the sample.

Approximately half of the samples were ST69 and its single-locus variants forming the ST69 Complex, which represent 148 isolates (64.6%) and formed a distinct monophyletic clade within the tree, indicating a strong degree of genetic conservation. In contrast, the other 81 isolates were distributed across different STs, including ST394 Complex (*n* = 19), ST31 Complex (*n* = 18), ST501 (*n* = 9), and ST7083 (*n* = 6), as well as various singletons. It is noteworthy that all but two of the strains in the ST69 Complex and all of the strains in the ST394 Complex are O77g:H18, while the ST31 Complex contains different serotypes.

Both human and non-human reservoirs were represented in the source of isolates. The majority of isolates (*n* = 107; 46.7%) were from human sources, followed by isolates from poultry (*n* = 46; 20.1%), livestock (*n* = 24; 10.5%), and environmental samples (*n* = 17; 7.4%). Additional sources were food (*n* = 8), companion animals (*n* = 6), wild animals (*n* = 9), and animal feed (*n* = 4). Notably, ST69 was found in environmental samples, livestock, and poultry in addition to human cases, indicating the possibility of interspecies transmission and persistence in non-clinical niches.

The most represented serotype was O77g:H18 because the database was constructed around that serotype and was found in 184 isolates (80.3%). One could notice that O77g:H18 strains belong to multiple STs (multiple lineages). This serotype is, however, strongly associated with the ST69 lineage as 129 isolates are both O77g:H18 and ST69. Even though the construction of the data set may have been biased toward ST69, 62% of all O77g:H18 strains in EnteroBase (access date: 20 May 2025) belong to ST69. Other serotypes were also included, in particular, O77g:H45 (*n* = 18), O77g:H1 (*n* = 10), and O77g:H34 (*n* = 9), but they were more distributed across different STs and branches of the phylogenetic tree.

**Fig 2:**
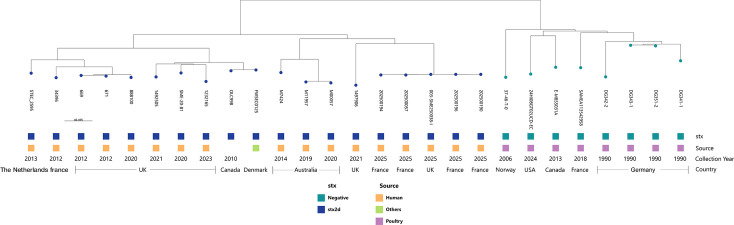
Subtree of the neighbor-joining tree showing only the “outbreak cluster” and the closest clade.

Around three-quarters of the data set was *stx*-negative (*n* = 174; 76%), suggesting a loss of the *stx*-prophage or that they never acquired it. Among *stx*-positive isolates, the most represented are isolates containing *stx2d* (*n* = 28), *stx2a* (*n* = 11), and *stx1a* (*n* = 9). A minority of isolates were found to contain both *stx1* and *stx2*, such as *stx1a*/*stx2a* (*n* = 4) and *stx1c*/*stx2a* (*n* = 1). Some rare *stx2* subtypes were found, like *stx2j* and *stx2l*, each detected in a single isolate. Within ST69, most isolates were *stx*-negative, but some harbor *stx2d* (*n* = 22) and, interestingly, were isolated from human clinical samples. A subtree of the neighbor-joining tree was extracted to study the cluster containing the clinical strains (*n* = 27) herein referred to as “outbreak cluster” (Fig. 2) and compare it to the closest genomic clade. This cluster was composed of a majority of human isolates ST69, O77g:H18, and *stx2d*. There are also two isolates from a different source, one from food and the other one from an unknown source. The four isolates selected among the 2025 cases in France were clustered together with isolate 003-SME250038-I. The latter isolate was isolated from a clinical case from Scotland; the diseased patient ordered a French cheese through the internet. Most of the isolates in that lineage were isolated from clinical samples. The closest cluster was composed of isolates from poultry sources (and thus referred to as the “poultry cluster”), originating from different countries in Europe and North America between 1990 and 2024, and interestingly, they were ST69, O77g:H18 but *stx*-negative. It is worth noting that French STEC O77g:H18 strains (ECA64, 98HMPL325) isolated from dairy products in the 1990s belong to a different ST or genetic lineage from the “outbreak cluster” (Fig. 1).

### Analysis of *stx*-encoding prophage

Complete *stx2d*-prophage sequences were obtained from the complete genomes of the Australian clinical strains M00057, M11957, and M7424. These strains are the only strains from the outbreak cluster with a high-quality genome available at the time. All three prophages (45.8 kbp in length) are inserted at the SerU locus, which is an unusual insertion site for *stx2d*-encoding phages (36, 40). By examining the *stx2d*-carrying contigs, the same insertion site was identified in the draft genomes of all the other *stx2d*-positive O77g:H18 strains of the “outbreak cluster” except for strains 003-SME250038-I and 202500190 for which the prophage sequences were fragmented and the insertion site could not be determined. The same insertion site was also identified in the *stx2d*-positive strains outside the “outbreak cluster” that carried the exact same *stx2d*-C165-O73-02 variant (PNUSAE109715, CFIAFB20180580, and 202312032). The insertion sites for the strains outside the “outbreak cluster” carrying other *stx2d* variants could not be identified, except for the French strain 98HMPL325, for which the insertion site was SerT.

**Fig 3:**
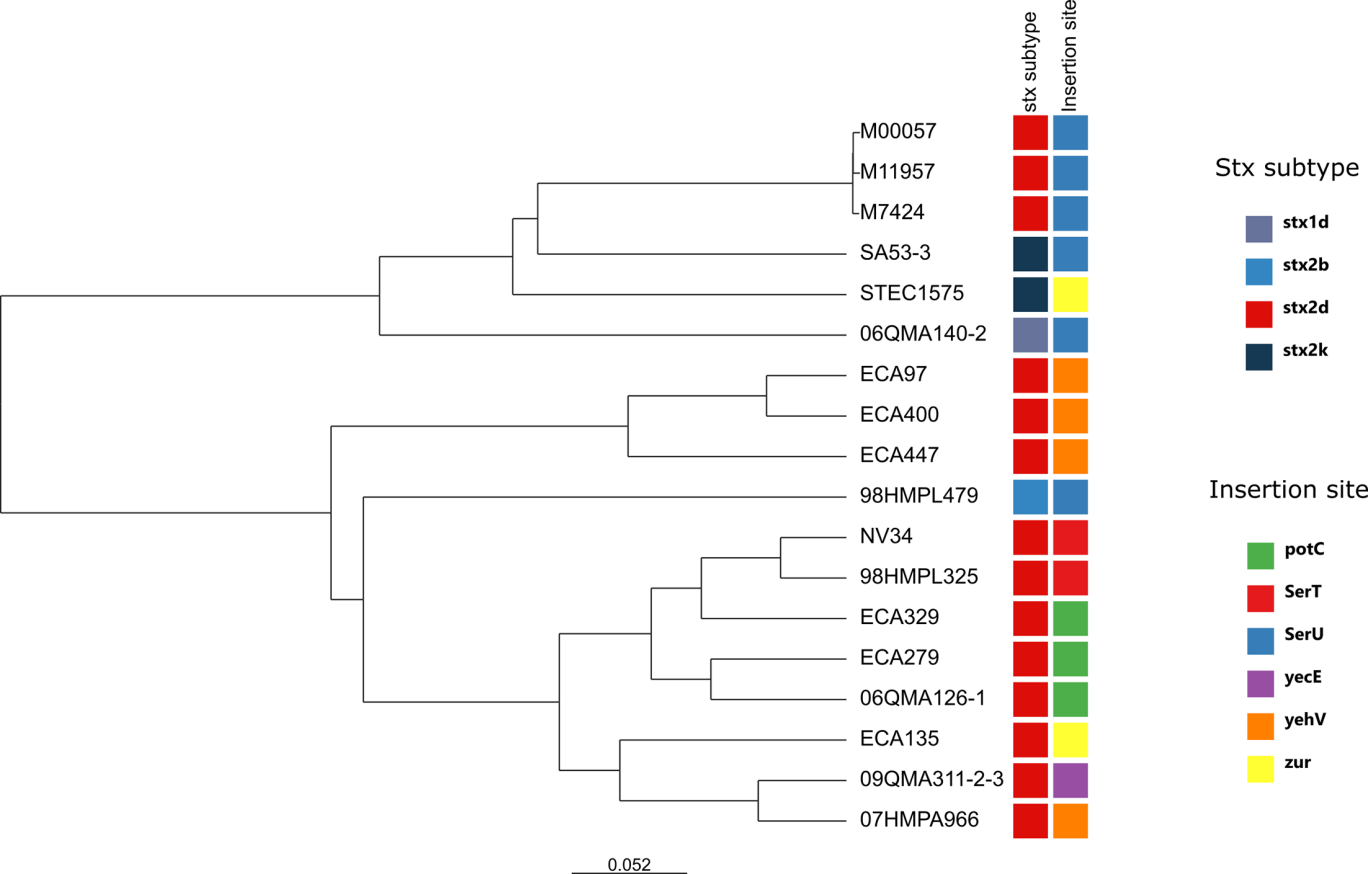
k-mer based UPGMA tree (*k*-mer = 15) of *stx*-phages showing the *stx* subtypes and the insertion site of the *stx* prophages.

BLASTn analysis of the *stx2d*-encoding prophage of the Australian clinical STEC O77g:H18 strain revealed the absence of homologous phage in the NCBI database. When compared with other *stx2d*-encoding phages of bovine origin (File S3), these phages showed limited similarities. However, there is greater genetic proximity to other *stx*-phages inserted at the SerU site, and in particular to *stx2k* phages inserted at SerU (Fig. 3; File S5).

### Analysis of virulence genes

To further study the genetic difference among *E. coli* O77g:H18 lineages from the “outbreak cluster” and “poultry cluster”, we analyzed the prevalence of 92 VGs shown in File S6.

A few differences in the virulence gene composition between the “outbreak cluster” and the “poultry cluster” were observed. The *sitA*, *iutA*, *iucABCD*, *iha*, and *iss* genes were detected in all strains of the “poultry cluster,” while they were absent in all strains of the “outbreak cluster” except for the French clinical strain 34396 identified in 2012 (Enterobase assembly barcode ESC_WB8404AA_AS), which was positive for *sitA*, *iutA*, and *iucABCD*. The hemolysin encoding gene *hlyF* (one marker of the pS88 plasmid [41]) was present in half the strains of the “poultry cluster” and absent in all strains of the “outbreak cluster.”

Few VGs were present with various degrees of prevalence in strains of the “outbreak cluster” only. Hence, the cytolethal distending toxin encoding gene (*cdt-V*) was only detected in all strains of the “outbreak cluster.” The gene encoding a putative enterotoxin (*elt*, HL-IIA) carried by an IncFIA/FIB plasmid (File S7) was detected in 16 out of the 19 strains of the “outbreak cluster,” including the French outbreak strain and the three Australian clinical strains M00057, M11957, and M7424. This putative enterotoxin appears extremely rare, as a BLASTn search for the *elt* gene shows that it is not found outside of the few O77g:H18 strains referenced here. Differences in the prevalence between the sub-units A and B of the putative enterotoxin (HL-IIA) may be due either to difficulties to assemble the IncFIA/FIB plasmid and the quality of the assemblies available in the public databases or to a truncated gene. Apart from these genes, the distribution of all the other VGs did not display any cluster specificity. Notably, the Long polar fimbriae (*lpfA*), type 1 fimbriae (*fimH*), and the *E. coli* hemolysin E (*hlyE*) encoding genes were present both in the “outbreak cluster” and the “poultry cluster.” On the contrary, genes involved classically in STEC attachment to the gut mucosa (*eae* and *aggR*) as well as the EHEC enterohemolysin gene (*ehxA*) were absent in the two clusters.

We next detected the resistance genes related to various classes of antibiotics among *E. coli* O77g:H18 lineages of the “outbreak cluster” and “poultry cluster” (File S6). The strains of the “poultry cluster” carried few antibiotic resistance genes. Regarding the clinical strains of the “outbreak cluster,” they were all negative for all resistance genes, except the French clinical strain 34396 identified in 2012. Indeed, this STEC O77g:H18 strain carried antibiotic resistance genes, including *bla*_TEM_, *strB* (aph(6)-Id), *ermB*, *mphB*, and *sul1*.

### PCR assays on cheese samples

The cheese sample collected from the suspected outbreak batch tested positive for both *stx2*, *wzy*_O77_, and HL-IIA subunits A and B gene targets. In contrast, among the 45 raw milk cheeses collected outside of the outbreak context, 36 samples were positive for *stx2*; 19 out of these 36 samples were positive for both *stx2* and wzy_O77_, but none for HL-IIA-A or HL-IIA-B (data not shown). These results confirm that the simultaneous detection of the three genetic markers is a strong indicator of cheese contaminated by the French outbreak STEC O77g:H18 strain.

## DISCUSSION

*E. coli* is a highly diverse and widely distributed bacterial species present in natural environments. Some *E. coli* strains possess the ability to cause a wide range of diseases, from intestinal to extra-intestinal infections (42). Strains responsible for the latter are classified as extra-intestinal pathogenic *E. coli* (ExPEC). Among them, *E. coli* O77g:H18 belonging to the ST69 complex is mainly associated with urinary tract infections (43). ExPEC strains share mutual virulence traits with APEC strains (42, 44, 45). Accordingly, *E. coli* O77g:H18-ST69 is frequently found in poultry (20). On the contrary, it is more rarely associated with intestinal infection in humans. Intestinal infections leading to bloody diarrhea and HUS have been reported in only very few sporadic cases with strains of *E. coli* O77g:H18-ST69 carrying either the *stx2a* or *stx2d* genes.

In 2025, the French National Reference Center for *Escherichia coli*, *Shigella*, and *Salmonella* detected several cases of adults infected by STEC serotype O77g:H18, *stx2d*-positive, *eae*-negative, ST69, and HC5_326896 (EnteroBase HierCC-cgMLST scheme). Epidemiological investigations conducted by Santé publique France linked this outbreak to the consumption of unpasteurized cheese from the same manufacturer. However, identifying the contaminated dairy farms proved to be challenging due to complex source tracing. Indeed, this STEC O77g:H18 strain harbored no resistance to antimicrobial agents and did not grow on STEC medium or sorbitol MacConkey (SMAC) agar, which made it difficult to isolate. Also, as STEC O77g:H18 was negative for the *ehxA* gene, it could not be identified on washed sheep blood agar, looking for enterohemolysin-producing STEC. Clinical isolates were recovered by random testing of many colonies cultivated on non-selective media. In raw milk, the high background flora of gram-negative bacteria, including *Escherichia*, and the absence of permissive selective agar plates for the outbreak strain explained the difficulties in confirming the presence of this clone in the raw milk samples investigated by local laboratories in charge of routine testing.

Nevertheless, as bovines excrete STEC in their feces with a high occurrence, contamination of raw milk by bovine feces is obviously suspected to be the main source of contamination. However, birds could also disseminate STEC into environments and/or transmit STEC to other animals directly or indirectly. Examining wildlife living close to cattle and pig farms in Denmark revealed that cattle and a starling (*Sturnus vulgaris*) harbored identical STEC strains, implying a role of wild birds in STEC transmission (46). A similar observation was reported in the United States, where STEC O157:H7 recovered from starlings near several dairy farms at a distinct geographical location was genotypically indistinguishable from the STEC O157:H7 strains recovered from cattle in the dairy farms (47). In fact, transmission of STEC O157:H7 from STEC-positive birds (European starlings) to the STEC-negative birds and to cattle was demonstrated under experimental conditions (48). Therefore, this represents another plausible source of contamination. The phylogenetic analysis based on the data set of *E. coli* O77g:H18 strains designed in this study aimed to provide some indications to identify the source of the contamination in the context of this outbreak. This data set enabled us to identify the genetic relatedness between the French outbreak strain and strains issued from different countries and sources collected over a period of 85 years.

We showed that the French outbreak strain O77g:H18 is located within a cluster of strains, all isolated from human clinical cases between 2012 and 2025. These strains are all O77g:H18, ST69, *stx*2d-positive, *eae*-negative, including one clinical isolate derived from one HUS case in France (2012), and three clinical isolates derived from HUS cases in Australia (2014, 2019, and 2020). The French STEC O77g:H18, ST69, *stx*2d-positive outbreak strain of 2025 has therefore already been associated with one HUS case in 2012 in France, and this is not a new lineage. Genetic analysis showed that the *stx*2d phage of this strain has no known homolog in GenBank. Comparison of the *stx2d* phage with other *stx2d* phages from French STEC strains of bovine origin (dairy products and minced meat) showed little similarity, suggesting a different niche for this specific *stx*-phage. This hypothesis is supported by the observation that *stx2d*-positive O77g:H18 strains from our data set isolated from dairy products in France in the 1990s both belong to a different genetic lineage (ST663) and harbor a different *stx2d* prophage from the outbreak strain. Also, our qPCR analyses of a small number of raw milk cheeses with *wzy*_O77_ and HL-IIA confirm the presence of O77g strains and the absence of the putative enterotoxin in these samples. Therefore, although this serotype appears to circulate in cattle, this particular strain is unusual.

The closest cluster to the outbreak strain contains eight *stx*-negative O77g:H18, ST69 *E. coli* strains, all isolated from poultry between 1990 and 2024 in Europe and North America. Differences in virulence gene composition between the “outbreak cluster” and the “poultry cluster” suggest that the two clusters have a common ancestor but have likely diverged over time. The “outbreak cluster” is characterized by STEC O77g:H18, ST69, *stx*2d-positive strains containing an IncFIA/FIB plasmid carrying a gene encoding a putative enterotoxin (HL-IIA) that is not found in any other *E. coli* strain tested in GenBank. We have developed qPCR assays specific for the putative enterotoxin gene (*elt*) and the *wzy* gene of O-group O77g. It could be noticed, however, that O77g belongs to a group of serogroups having the same O-antigen cluster: O17, O44, O106, O73, and O77 (15, 16). Therefore, the qPCR test designed in this study to target the *wzy*-O77 gene is able to detect without any distinction these five O-groups (data not shown). Nevertheless, when combined with PCR assays targeting the *stx*2 gene and the enterotoxin gene, it allowed refining the detection scheme and making it easier to rapidly screen suspect food samples for the French STEC O77g:H18, ST69, *stx*2d outbreak strain. Hence, we detected PCR-positive signals with these three PCR tests when testing one unpasteurized cheese sample that was issued from the batch recalled by the French general directorate of food of the French ministry for agriculture and food.

In conclusion, despite the fact that it remains difficult to fully define human pathogenic STEC or identify virulence factors for STEC that absolutely predict the potential to cause human disease (49), we confirm here that the virulence genes profile or the virulence genes repertoire, rather than serotype, is much more meaningful in predicting the pathogenicity potential of uncharacterized STEC O77g:H18 strains. Indeed, it is well known that some *stx* subtypes like *stx2d* are most frequently implicated in causing severe human illness, including HUS (50). In this study, we showed that *E. coli* O77g:H18-ST69 strains carrying *stx2*d and a plasmid-borne enterotoxin gene (*elt*) have an enhanced pathogenicity potential. This clone could contaminate raw milk, persist in unpasteurized cheese, and induce HUS in adults. Such a rare and atypical STEC strain associated with sporadic HUS cases can emerge from different ecological niches. In the present case, the phylogenetic analysis suggested that the epidemic French clone of STEC O77g:H18 probably had a common ancestor with strains originated from birds or poultry.

## Data Availability

Assemblies of the sequenced strains have been deposited in the European Nucleotide Archive database (ENA) under BioProject PRJEB89238. The Enterobase data set can be accessed at https://enterobase.warwick.ac.uk/species/ecoli/search_strains?query=workspace:145591.

## SUPPLEMENTAL MATERIAL

File S1 aem.02449-25-s0001.csv Listing of the 229 *E. coli* O77g strains and their corresponding metadata.

File S2 aem.02449-25-s0002.csv Python script used during cgMLST analysis.

File S3 aem.02449-25-s0003.pdf *stx* phages analysis.

File S4 aem.02449-25-s0004.pdf Primer and probe sequences.

File S5 aem.02449-25-s0005.xlsx Blast (A) and Mauve (B) comparisons of *stx*-phages.

File S6 aem.02449-25-s0006.xlsx Virulence and AMR gene analysis.

File S7 aem.02449-25-s0007.pdf Representation of the IncFIA/FIB plasmid of strain M7424.

ASM does not own the copyrights to Supplemental Material that may be linked to, or accessed through, an article. The authors have granted ASM a non-exclusive, world-wide license to publish the Supplemental Material files. Please contact the corresponding author directly for reuse.
