# Supplementary material for: Analysis of the genetic and phylogenetic context of Escherichia coli O77g:H18 associated with clustered cases of HUS in France in 2025
Source: Appl Environ Microbiol. 2026 Apr 24;92(5):e02449-25. doi: 10.1128/aem.02449-25 (PMC13188871; doi:10.1128/aem.02449-25)
Supplement: File S4 — Primer and probe sequences. [file aem.02449-25-s0004.pdf]

Primers and probes

Name	Primers and probes sequences (5'-3')
HL-IIA-B-F	TGTTGTCTCTGAAAGTGGAGG
HL-IIA-B-R	CACTTATTCCTTATTTCTATTGACCA
HL-IIA-B-Taq	TTCGGTTGCACACAGACTAACTGGCATAC
HL-IIA-A-F	TGATCATGCAACAGGTACTCC
HL-IIA-A-R	GAACCAAGCACACCATTTACATC
HL-IIA-A-Taq	ATCGACAACCAGGACGTTAACTAGCGCA
wzy_O77-F	GATCCCATCCTGATGATTGCA
wzy_O77-R	CGTGAAGGGTACCAAATCGATT
wzy_O77-Taq	ATCAAACAAATTCTGCATCCCCCCAAGA
