# Supplementary figures and images for: Analysis of the genetic and phylogenetic context of Escherichia coli O77g:H18 associated with clustered cases of HUS in France in 2025
Source: Appl Environ Microbiol. 2026 Apr 24;92(5):e02449-25. doi: 10.1128/aem.02449-25 (PMC13188871; doi:10.1128/aem.02449-25)

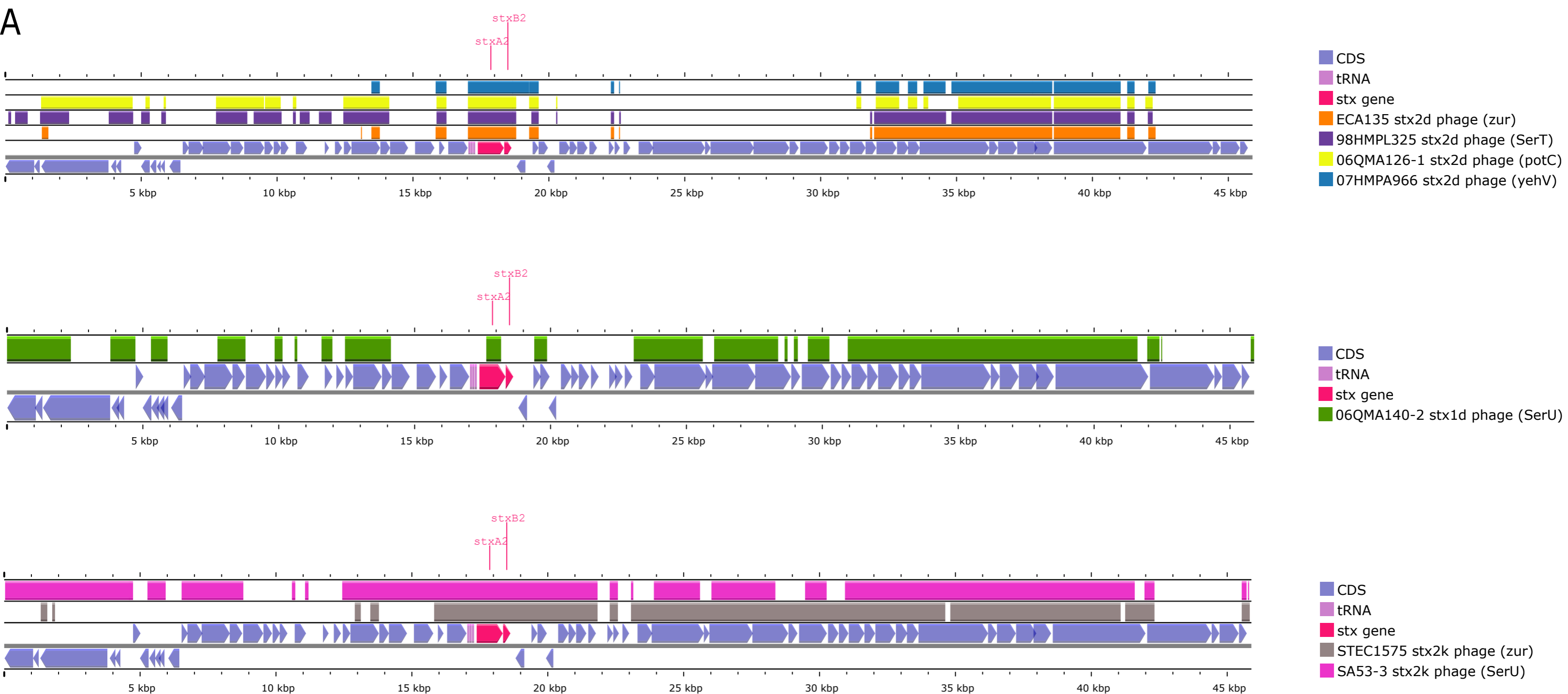

M00057 stx2d phage

**B**

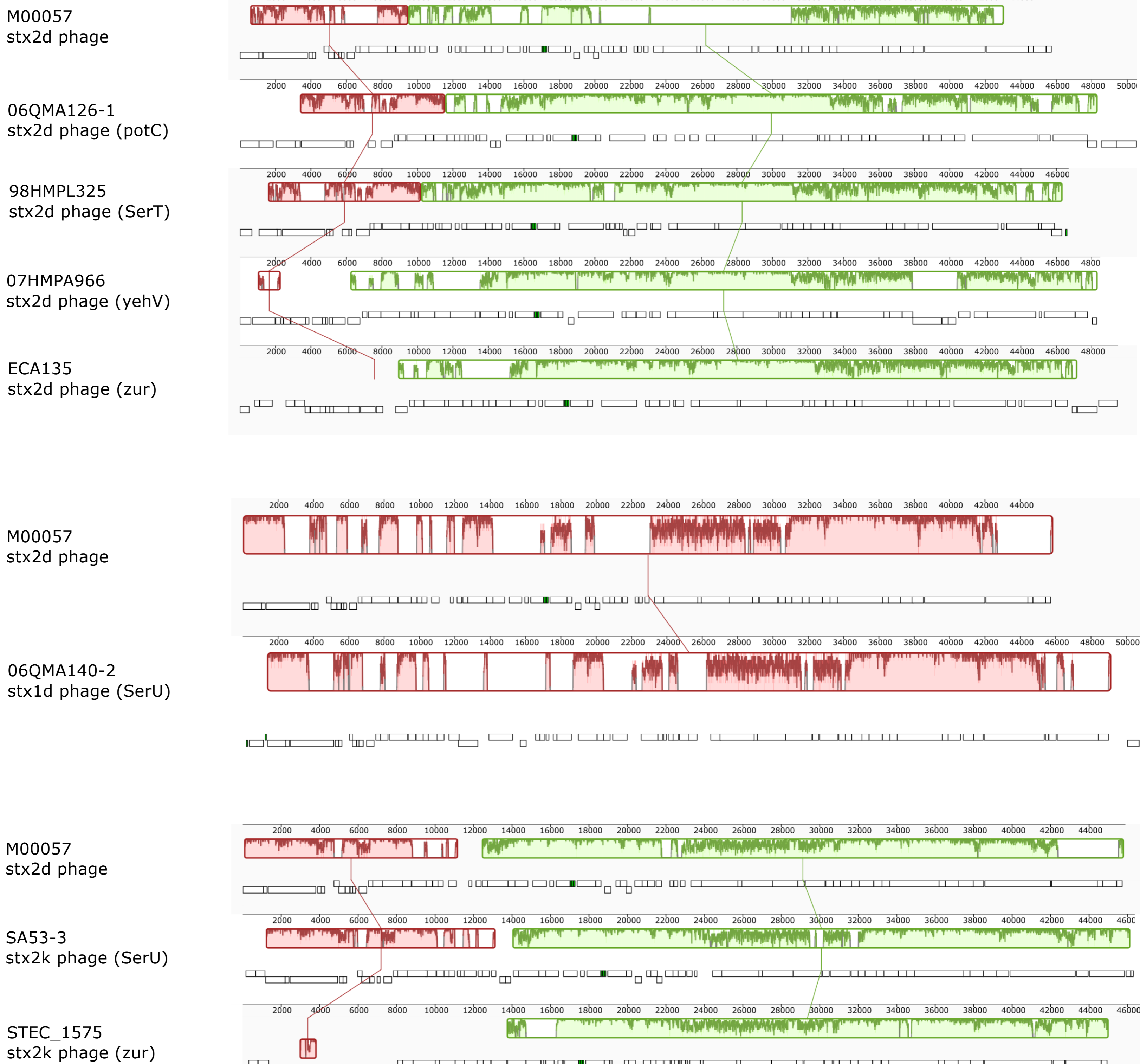

Supplement: File S7 — Representation of the IncFIA/FIB plasmid of strain M7424. [file aem.02449-25-s0007.pdf]
